# CXCR4 mediates matrix stiffness-induced downregulation of UBTD1 driving hepatocellular carcinoma progression via YAP signaling pathway

**DOI:** 10.7150/thno.44789

**Published:** 2020-04-27

**Authors:** Nan Yang, Tianxiang Chen, Liang Wang, Runkun Liu, Yongshen Niu, Liankang Sun, Bowen Yao, Yufeng Wang, Wei Yang, Qingguang Liu, Kangsheng Tu, Zhikui Liu

**Affiliations:** 1 Department of Infectious Diseases, the First Affiliated Hospital of Xi'an Jiaotong University, No. 277 Yanta West Road, Xi'an 710061, China; 2 Department of Hepatobiliary Surgery, the First Affiliated Hospital of Xi'an Jiaotong University, No. 277 Yanta West Road, Xi'an 710061, China

**Keywords:** C-X-C chemokine receptor 4, ubiquitin domain-containing protein 1, hepatocellular carcinoma, Yes-associated protein, matrix stiffness

## Abstract

**Rational**: Increasing evidence indicates that the physical environment is a critical mediator of tumor behavior. Hepatocellular carcinoma (HCC) develops in an altered biomechanical environment, and increased matrix stiffness is a strong predictor of HCC development. C-X-C chemokine receptor type 4 (CXCR4) is known to trigger HCC progression. However, CXCR4 as a mediator of mechanical cues in HCC is not well characterized.

**Methods**: qRT-PCR, Western blot and IHC were used to detect the CXCR4 expression in different matrix stiffness gels. MTT was used to measure the cell proliferation of HCC cells. Immunoblotting was used for detection of epithelial-to-mesenchymal transition (EMT) and stemness on the matrix stiffness. Immunofluorescence (IF) was used to detect the nuclear location in HCC cells. IP was used to show the interaction between YAP, UbcH5c and β-TrCP.

**Results**: We identified CXCR4 as a critical intracellular signal transducer that relays matrix stiffness signals to control mechano-sensitive cellular activities through ubiquitin domain-containing protein 1 (UBTD1)-mediated YAP signaling pathway. We found that CXCR4 expression was remarkably up-regulated in HCC cells with increasing matrix stiffness and mediated proliferation, epithelial to mesenchymal transition, and stemness. Mechanistically, matrix stiffness acts through CXCR4 to decrease the levels of UBTD1, which is involved in the proteasome-dependent degradation of YAP, a major cell mechano-transducer. UBTD1 interacted with components of the YAP degradation complex and promoted the interaction between YAP and its E3 ubiquitin ligase β-TrCP. UBTD1 knockdown decreased YAP ubiquitylation and resulted in the activation of YAP-targeted genes and YAP downstream signaling. Downregulation of UBTD1 in HCC tissues correlated with malignant prognostic features and overall survival. Finally, luteolin, a natural product, suppressed matrix stiffness-induced biological effects and CXCR4-mediated YAP signaling pathway in HCC cells.

**Conclusion**: Our findings reveal CXCR4 as a molecular switch in mechano-transduction, thereby defining a mechano-signaling pathway from matrix stiffness to the nucleus.

## Introduction

Hepatocellular carcinoma (HCC) is the fifth most common malignancy and the second most frequent cause of cancer-related mortality worldwide 1. Clinically, the majorities of HCCs develop and progress in the context of advanced liver fibrosis or cirrhosis 2-4. Hepatic fibrosis is one of the major risk factors for developing HCC. Recently, ultrasound elastography (FibroScan) confirmed that liver stiffness measurement could be applied as a non-invasive predictor in the diagnosis of fibrosis, risk assessment of HCC development, and prognosis 5, 6. It has been reported that changes in biochemical and physical properties of the cellular microenvironment accompany the occurrence and development of chronic liver disease and fibrosis 7. Liver fibrosis was pathologically correlated with increased rigidity of the extracellular matrix (ECM), involving multiple signal transduction pathways that regulate HCC development 8, 9. However, the underlying molecular mechanisms of matrix stiffness-induced HCC development remain elusive.

During tumor progression, increased ECM deposition and cross-linking result in increased tumor stiffness. Cells recognize and respond to the mechanical stiffness of the ECM by exerting contractile force and sensing counter-tension through mechano-cellular systems 10. Cellular stiffness sensing relies upon intracellular tension, which is determined by a dynamic equilibrium between forces generated by the contractile cytoskeleton and elastic resistance provided by the ECM 11. Matrix stiffness affects the biological behavior of cancer cells, regulates cancer-related genes or non-coding RNA expression, and contributes to tumor progression, including invasion, metastasis, and stemness characteristics 12-15. Schrader et al. reported that matrix stiffness modulates dormancy, proliferation, and chemotherapeutic response in HCC cells with the stiffness ranging from 1 to 12 kPa^16^. Pang et al. demonstrated that substrate stiffness promotes latent TGF-β1 activation in HCC 17.

Chemokine receptors are important modulators of cell trafficking and adhesion 18, and their role in HCC development and progression is under intense investigation. Among them, C-X-C chemokine receptor type 4 (CXCR4) was shown to be involved in HCC cell proliferation, metastasis, and cell dissemination by interacting with its ligand SDF-1^19^-^21^. It was also reported that CXCR4 antagonism reduces cardiac fibrosis and improves cardiac systolic performance in dilated cardiomyopathy 22. CXCR4 density, spatial organization, and matrix stiffness are paramount to achieve strong binding in breast cancer 23. Although gene expression analysis showed overexpression of CXCR4 in HCC, its involvement in the development of HCC in response to mechanical cues remains unclear.

Ubiquitin domain-containing protein 1 (UBTD1) is highly evolutionarily conserved and has been described to interact with E2 enzymes of the ubiquitin-proteasome system 24, 25. UBTD1 induces cellular senescence through increasing the stability of p53 protein and promoting the degradation of Mdm2 protein in gastric cancer 26. Furthermore, depletion of UBTD1 drastically affects the mechanical properties of epithelial cancer cells via RhoA activation and strongly promotes aggressiveness 27. On a stiff matrix, UBTD1 expression is regulated by cell-cell contacts, and the protein is associated with β-catenin at cell junctions. Also, YAP/TAZ functions as essential effectors of mechano-transduction to regulate cell proliferation and differentiation 28. Matrix stiffness mediates stemness characteristics by activating YAP in colorectal cancer cells 14. YAP protein also regulates the growth of human non-small cell lung cancer in response to matrix stiffness. When cells are shifted from stiff to soft matrices, YAP/TAZ translocates from the nucleus to the cytoplasm and are thus inactivated 29. However, the mechanism of ECM stiffness on the Hippo/YAP pathway is unclear.

Here, we identified CXCR4 as a mechano-transducer to relay extracellular mechanical signals and induce UBTD1 down-regulation, which decreased YAP ubiquitylation degradation, resulting in the activation of YAP-targeted genes. We showed that UBTD1 was down-regulated in HCC and was associated with poor patient survival. UBTD1 promoted the interaction of YAP with its E3 ubiquitin ligase β-TrCP and decreased YAP ubiquitylation to trigger its activation and downstream signaling. Matrix stiffness acted through CXCR4 and its downstream UBTD1 to modulate a YAP/TAZ-mediated mechano-responsive signaling pathway. This new signaling axis provides novel insights to clarify how mechanical stimuli exerted their effects on cellular bio-response.

## Materials and Methods

### Polyacrylamide hydrogels with incremental stiffness

The mechanically tunable Collagen 1 (COL-1)-coated polyacrylamide gel was established as previously described 30. Briefly, polyacrylamide gels with different mechanical stiffness levels were prepared by mixing 10% acrylamide and 0.01% to 0.5% bis-acrylamide in a HEPES-buffered solution supplemented with 10% ammonium persulfate (APS) and TEMED. Subsequently, the formed gels were further cross-linked and coated with 0.1 mg/mL COL-1 solution (BD) suitable for cell culture.

### Clinical tissues and cell culture

HCC tissues and matched adjacent non-tumor tissues were obtained from our hospital. No patients received preoperative chemo- or radiotherapy before surgery. Written informed consent was obtained from each patient. The human HCC cell lines (Hep3B and Huh7) were obtained from the Chinese Academy of Sciences (Shanghai, China). The cells were cultured in Dulbecco's Modified Eagle's Medium (DMEM; Invitrogen, Carlsbad, CA, USA) supplemented with 10% fetal bovine serum (Gibco, GrandIsland, NY, USA), 100 units/mL ampicillin, and 100 μg/mL streptomycin at 37˚C with 5% CO_2_. Luteolin was purchased from Dalian Meilun Biology Technology Co., Ltd.

### Immunohistochemical staining (IHC)

Immunohistochemistry was performed on paraformaldehyde-fixed paraffin sections^31^, ^32^. CXCR4 (Cell Signaling Technology, #97680), UBTD1 (Invitrogen, HPA034825), and Ki67 (#9449) (1:100, Cell Signaling, Danvers, MA, USA) antibodies were used in immunohistochemistry using a streptavidin peroxidase-conjugated (SP-IHC) method. Immunohistochemistry was performed as previously reported^33^.

### Immunofluorescence (IF)

HCC cells were fixed with 4% paraformaldehyde and permeabilized using 0.2% Triton X-100. Then the fixed cells were incubated with the YAP (1:200, Cell Signaling, Danvers, MA, USA) primary antibody. The secondary antibody was an Alexa Fluor-conjugated IgG (Invitrogen, Carlsbad, CA, USA). Fluorescence confocal images were captured using an LSM 5 Pascal Laser Scanning Microscope (Zeiss Germany, Oberkochen, Germany) with a ×40 lens and Laser Scanning Microscope LSM PASCAL software (version 4.2 SP1).

### Western blot analysis

Total protein was extracted from HCC cells, and 20 μg of isolated proteins were separated by 10% SDS-PAGE, transferred onto a PVDF membrane (Bio-Rad Laboratories, Hercules, CA, USA), and probed with the respective primary antibodies (1:1000, Cell Signaling, Danvers, MA, USA) for overnight. Subsequently, the membranes were incubated with the HRP-conjugated goat anti-mouse or anti-rabbit IgG antibody (ZSGB-BIO, China). Protein bands were visualized using an enhanced chemiluminescence kit (Amersham, Little Chalfont, UK).

### MTT assay

Cells were seeded in 96-well plates, and the viability of cells was measured by the MTT assay. Briefly, 20 μL of MTT working solution (5 mg/mL) was added into each well and incubated at 37°C for 4 h. Then the supernatants were removed, and the resultant MTT formazan was dissolved in 100 μL of DMSO. The absorbance was measured at the wavelengths of 490 nM.

### YAP degradation assay

YAP protein stability on matrix stiffness was analyzed by WB in the presence of a protein synthesis inhibitor cycloheximide (CHX). In brief, CHX (50 μg/mL) was added to the cell culture medium for indicated times and cells were harvested for protein extraction. At each time point, cells were washed with cold PBS and lysed in RIPA buffer. Equal amounts of total protein per time point were then loaded onto a PAGE gel for YAP WB.

### *In vivo* assay

4-6-week-old male BALB/c nude mice (Centre of Laboratory Animals, The Medical College of Xi'an Jiaotong University, Xi'an, China) were randomized into two groups (n=5). The transfected cells (1×10^6^) were mixed in 150 µL of Matrigel and were inoculated subcutaneously into the flanks of one group of nude mice; the other group received transfected cells (1×10^6^) via tail vein injections for the establishment of the pulmonary metastatic model. The tumor volume for each mouse was determined by the following formula: tumor volume = length × width × width/2. After 3 weeks, the mice were sacrificed by cervical dislocation under anesthesia with ether and the xenograft tumor tissue was explanted for examination. All *in vivo* protocols were approved by the Institutional Animal Care and Use Committee of Xi'an Jiaotong University.

### Co-immunoprecipitation (Co-IP) assay

For the Co-IP assay, cells were lysed with lysis buffer. Cell lysates or control immunoglobulin (IgG). After extensive washing, precipitates were analyzed by Western blotting, which was performed using the standard protocol.

### Statistical analysis

Data were presented as the mean ± SD from at least three independent replicates. SPSS software, 16.0 (SPSS, Inc, Chicago, IL, USA) was used to conduct the analysis, and a two-tailed Student t-test was employed to analyze the differences between the two groups. Pearson's correlation analysis was used to analyze the correlation between the two indices. Differences were considered statistically significant at P < 0.05.

## Results

### Matrix stiffness affects HCC cellular behavior through CXCR4

**Figure 1:**
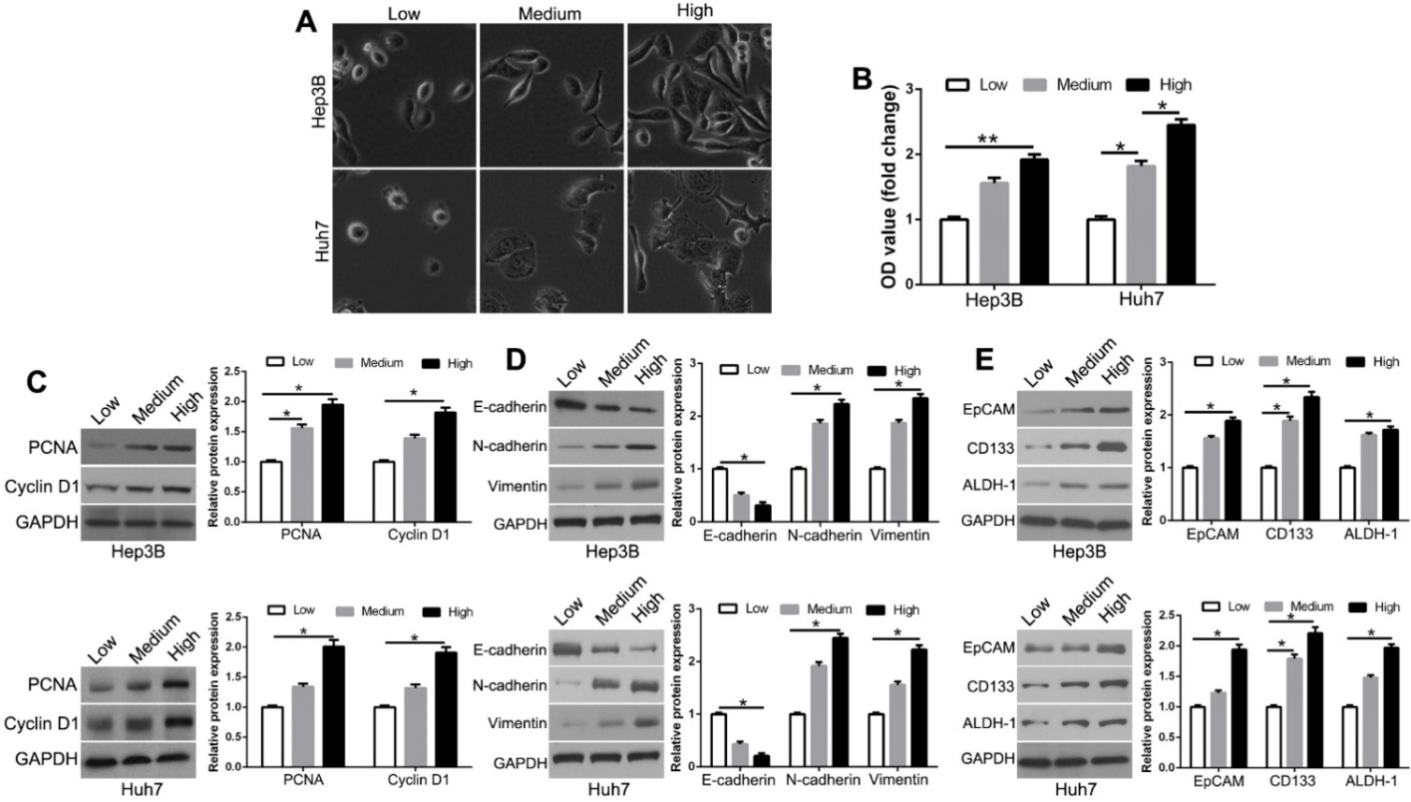
Increased matrix stiffness regulates HCC morphology, proliferation, EMT phenotype shift, and stemness characteristics of HCC cells. (A) Morphology changes of Hep3B and Huh7 cells cultured on gels of different stiffness. (B) Proliferation of Hep3B and Huh7 cells cultured on gels of different stiffness was measured using MTT assay. (C) PCNA and CyclinD1 expression in Hep3B and Huh7 cells on gels of different stiffness were determined by Western blotting. (D) Western blotting of whole-cell lysates showing expression of E-cadherin, N-cadherin, and vimentin in Hep3B and Huh7 cells cultured on different stiffness. (E) Increasing matrix stiffness remarkably upregulated the expression of CD133, EpCAM, and ALDH-1 in two HCC cells. *P < 0.05, **P < 0.01.

Increasing matrix stiffness constructed with mechanical gels was used to investigate the response of HCC cells. We used the low (1 kPa), medium (6 kPa), and high (12 kPa) matrix stiffness to represent the normal, fibrosis, and cirrhosis HCC tissue background, respectively. We observed the morphological changes of Hep3B and Huh7 cells from small and round to fully spread and outstretched on different stiffness platforms (Figure 1A). Compared to soft gels, stiff substrate promoted the proliferative activity (Figure 1B, P < 0.05). This was observed with the expression levels of proliferation-related markers, PCNA and Cyclin D1 (Figure 1C, P < 0.05). Matrix stiffness also increased the mesenchymal markers N-cadherin and vimentin and decreased the epithelial marker E-cadherin (Figure 1D, P < 0.05). Compared with soft gels, the stem cell markers EpCAM, CD133, and ALDH-1 were elevated in the cells on stiff gels (Figure 1E, P < 0.05). These results suggest that higher matrix stiffness enhances proliferation, epithelial to mesenchymal transition (EMT) phenotype, and stemness characteristics of HCC cells.

**Figure 2:**
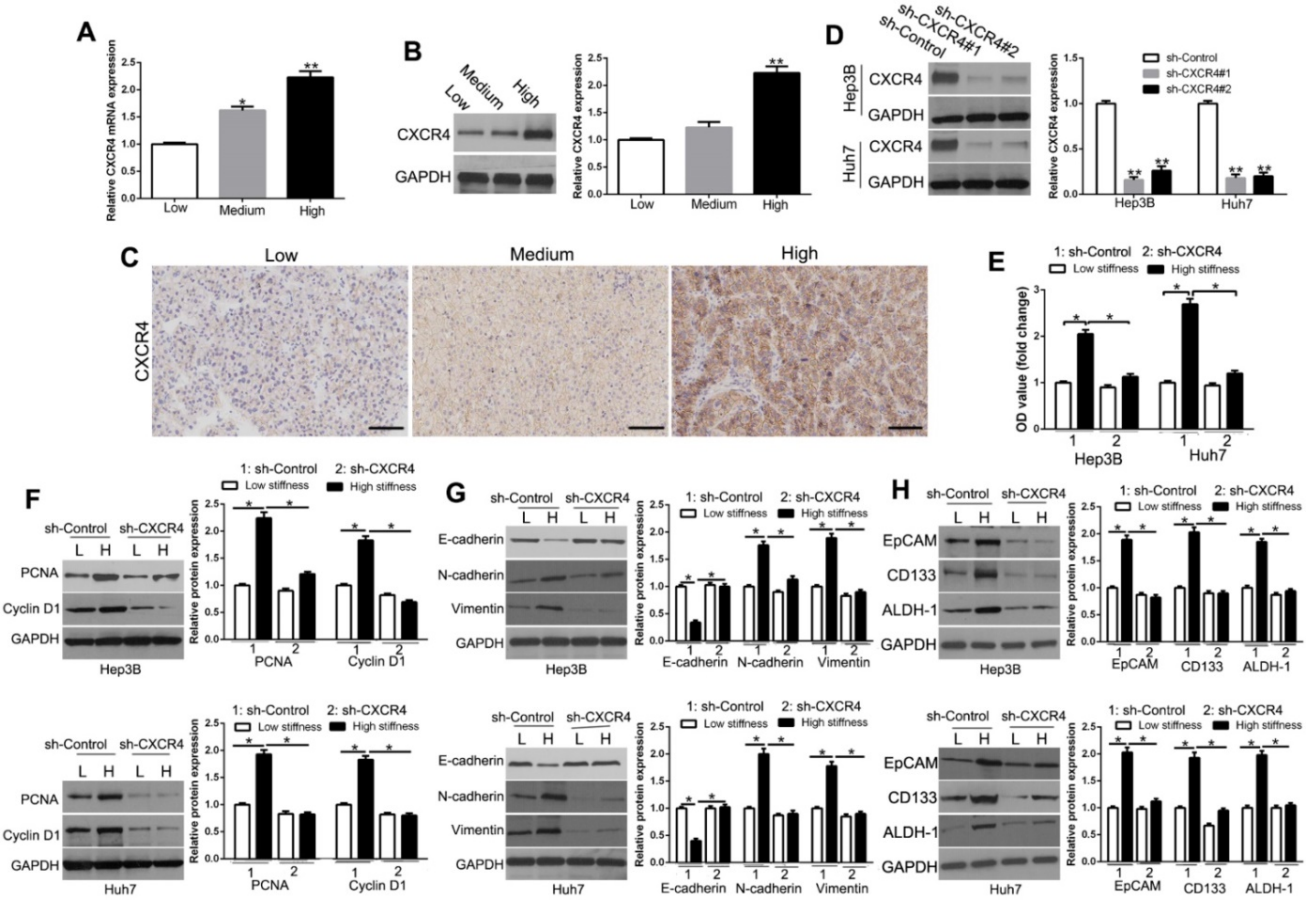
CXCR4 is involved in proliferation, EMT, and stemness characteristics induced by matrix stiffness. mRNA (A) and protein (B) were significantly up-regulated with increasing matrix stiffness. (C) Expression levels of CXCR4 in HCC tissues with normal (groups L, N=25), medium (group M, N=35), and high liver stiffness backgrounds (group H, N=46). (D) Hep3B and Huh7 cells transfected with shRNA were subjected to Western blotting for CXCR4 expression. (E) MTT revealed that stiffness-mediated HCC proliferation was abrogated by CXCR4 gene deletion. HCC cells seeded on low or high stiffness were transduced with NT shRNA or CXCR4 shRNA. Stiffness-mediated HCC (PCNA) and CyclinD1 expression (F), EMT (G) and stemness characteristics (H) were abrogated by CXCR4 knockdown. Bar: 70μM. *P < 0.05, **P < 0.01.

To examine the effect of matrix stiffness on CXCR4, we measured its transcriptional and translational levels on different cell culture platforms. We found that CXCR4 mRNA (Figure 2A, P < 0.05) and protein (Figure 2B, P < 0.05) were significantly up-regulated in Hep3B cells with increasing matrix stiffness. To confirm the CXCR4 expression level *in vivo*, we performed IHC staining on HCC tissues and found that CXCR4 and EMT-related proteins were highly expressed in HCC tissues with high and medium stiffness background as compared with that of normal stiffness background (Figure 2C, Figure S1, Figure S2), which was consistent with the results *in vitro*. The stiffness background of the extracellular matrix was confirmed by Sirius red and trichrome staining (Figure S3). To determine whether CXCR4 mediated the mechanical effects in HCC, we used shRNA to knockdown CXCR4 expression of HCC cells cultured on plastic plates (Figure 2D, P < 0.05). CXCR4 knockdown inhibited proliferation, EMT, and stemness on the stiff matrix (Figure 2E-H, P < 0.05). These results implied that CXCR4 acts as an essential regulator in cellular behavior-related gene expression as it pertains to the matrix stiffness environment.

### CXCR4 is involved in YAP activation and nuclear localization by matrix stiffness

**Figure 3:**
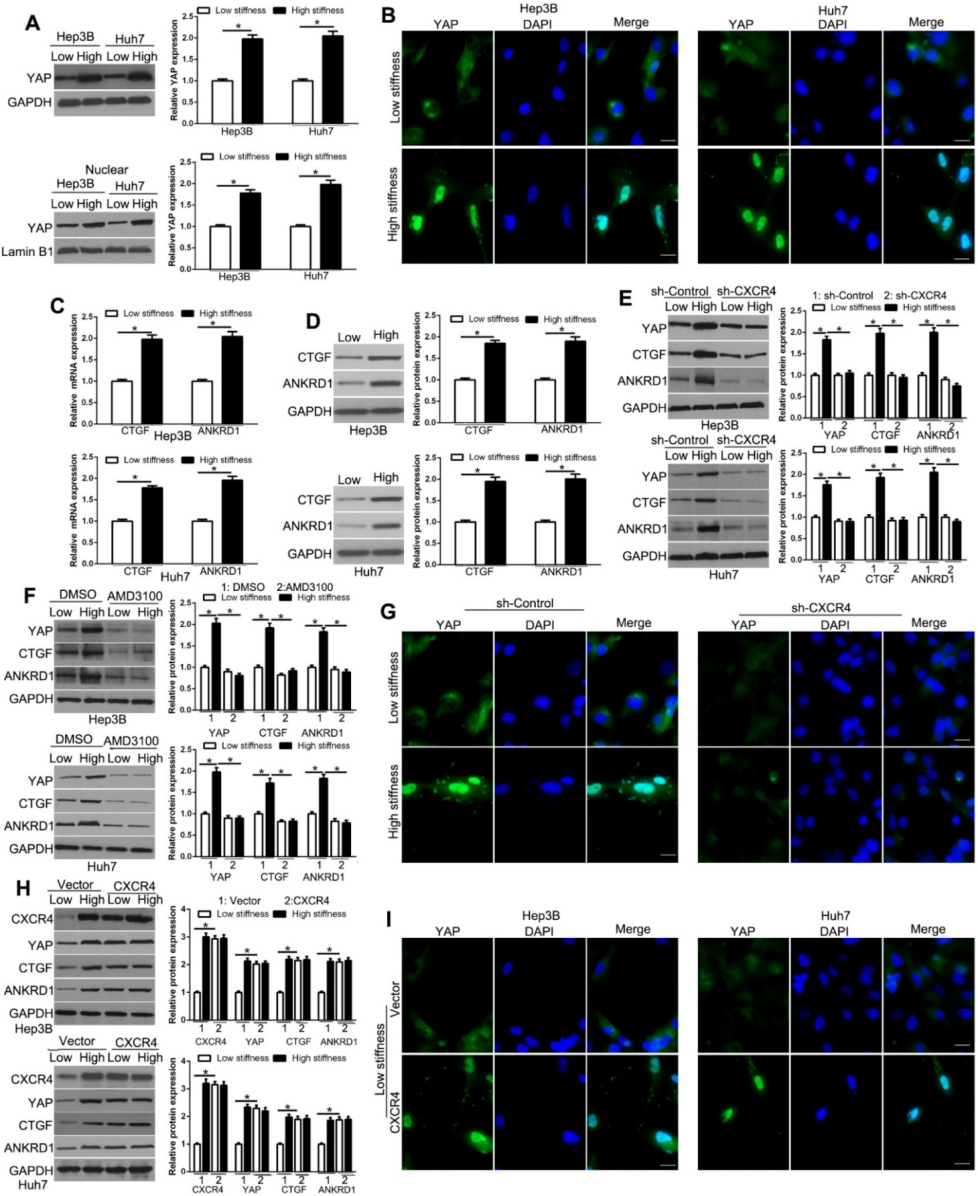
CXCR4 is involved in YAP activation and nuclear localization by matrix stiffness. Subcellular fractionation (A) and IF (B) assay were used to analyze stiffness-induced YAP nuclear accumulation. (C) RT-qPCR and WB (D) analysis of YAP target genes CTGF and ANKRD1 mRNA and protein levels were performed in Hep3B and Huh7 cells on low or high stiffness. Stiffness-mediated YAP nuclear accumulation and target gene expression measured by WB was abrogated by CXCR4 shRNA (E) or inhibitor AMD3100 (100 nM) (F). (G) Stiffness-mediated YAP upregulation and nuclear accumulation were abrogated by CXCR4 shRNA in Hep3B cells. (H, I) Hep3B and Huh7 cells transduced with CXCR4 overexpression vectors were seeded on low or high stiffness for WB and IF. CXCR4 led to YAP upregulation and nuclear accumulation on low stiffness. Bar:20μM.*P < 0.05.

The Hippo pathway effectors YAP and TAZ are regulated by mechanical cues and mediate cellular responses to ECM stiffness. YAP/TAZ is functionally redundant homologous transcriptional activators that can promote oncogenic transformations involving EMT 34, 35. IF and subcellular fractionation analyses showed that high matrix stiffness enhanced the YAP protein level and concurrent YAP nuclear accumulation in HCC cells compared to low stiffness (Figure 3A-B, Figure S4, P < 0.05). Also, the expression of the YAP target genes, CTGF and ANKRD1, were increased with stiffness, as determined by PCR and WB (Figure 3C-D, P < 0.05), confirming that YAP activity was affected by stiffness. We next used CXCR4 shRNA or inhibitor AMD3100 to test the role of CXCR4 in mechano-transduction-induced YAP expression in HCC cells. Our data showed that CXCR4 knockdown suppressed stiffness-mediated up-regulation of YAP and its target genes in HCC (Figure 3E, P < 0.05). Pharmacological inhibition of CXCR4 by AMD3100 also showed similar results (Figure 3F, P < 0.05). Furthermore, stiffness-mediated YAP nuclear accumulation was significantly suppressed by CXCR4 shRNA in Hep3B and Huh7 cells (Figure 3G, Figure S5).On the contrary, CXCR4 overexpression by retroviral transduction of cells on the soft gel led to upregulation of YAP and its target genes and YAP nuclear accumulation, which was similar to the effects of stiffness (Figure 3H-I). Thus, our data supported that stiffness-mediated YAP activation and nuclear accumulation require CXCR4.

### Matrix stiffness down-regulates UBTD1 in HCC cells and regulates YAP degradation by the ubiquitin-proteasome system independent of Hippo signaling

**Figure 4:**
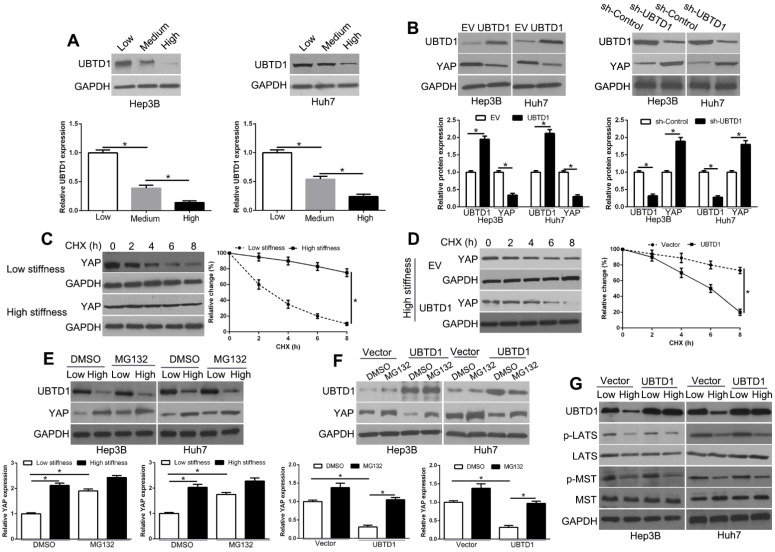
UBTD1 is down-regulated and regulates YAP degradation by the ubiquitin-proteasome system independent of the Hippo signaling pathway by matrix stiffness in HCC cells. (A) UBTD1 was significantly down-regulated with increasing matrix stiffness in HCC cells. (B) UBTD1 overexpression inhibited while UBTD1 knockdown promoted YAP expression in HCC cells. (C) YAP protein degradation in Hep3B was assessed by WB in the presence of CHX. (D) UBTD1 overexpression promoted the YAP degradation rate and was analyzed following treatment with CHX (50 μg/mL). (E) Low stiffness reduced the YAP protein level and its effect was reversed by a proteasome inhibitor MG-132 (20 μM). (F) MG132 reversed UBTD1 overexpression-induced YAP decrease. (G) Hippo signaling cascade LATS1/2 and MST1/2 phosphorylation was not affected by UBTD1 overexpression. *P < 0.05.

YAP plays an important central role in the Hippo pathway. The Hippo kinases LATS1/2 and MST1/2 phosphorylate YAP (S127) and inhibit nuclear translocation of YAP and the subsequent transcriptional program 36. To investigate the mechanisms that are involved in YAP signaling pathway activation, we used WB to measure UBTD1, which was structurally identified as an ubiquitin-like protein and interacts stoichiometrically with the UbcH5c ubiquitin-conjugating enzyme. The data showed that UBTD1 was decreased with the increasing matrix stiffness (Figure 4A, P < 0.05). Moreover, UBTD1 overexpression inhibited YAP expression on high stiffness while UBTD1 knockdown increased YAP expression on soft stiffness (Figure 4B, P < 0.05, Figure S6). When we treated cells with a protein synthesis inhibitor CHX, YAP protein levels on soft stiffness degraded faster than it did on high stiffness (Figure 4C, P < 0.05). Also, as shown in Figure 4D, UBTD1 overexpression markedly shortened the half-life of YAP. To confirm that UBTD1 was involved in the proteasome-dependent degradation of YAP, we treated the cells with the proteasome inhibitor (MG132) and found that inhibition of proteasomal degradation induced an increase in YAP protein levels (Figure 4E, P < 0.05). MG132 could also reverse the UBTD1 overexpression-induced YAP decrease (Figure 4F, P < 0.05). Besides, the Hippo signaling cascade LATS1/2 and MST1/2 phosphorylation was not affected by UBTD1 overexpression in cells (Figure 4G). These data suggested that UBTD1 affects YAP expression independent of the canonical Hippo pathway.

### UBTD1 promotes association between YAP and β-TrCP to induce YAP ubiquitylation

**Figure 5:**
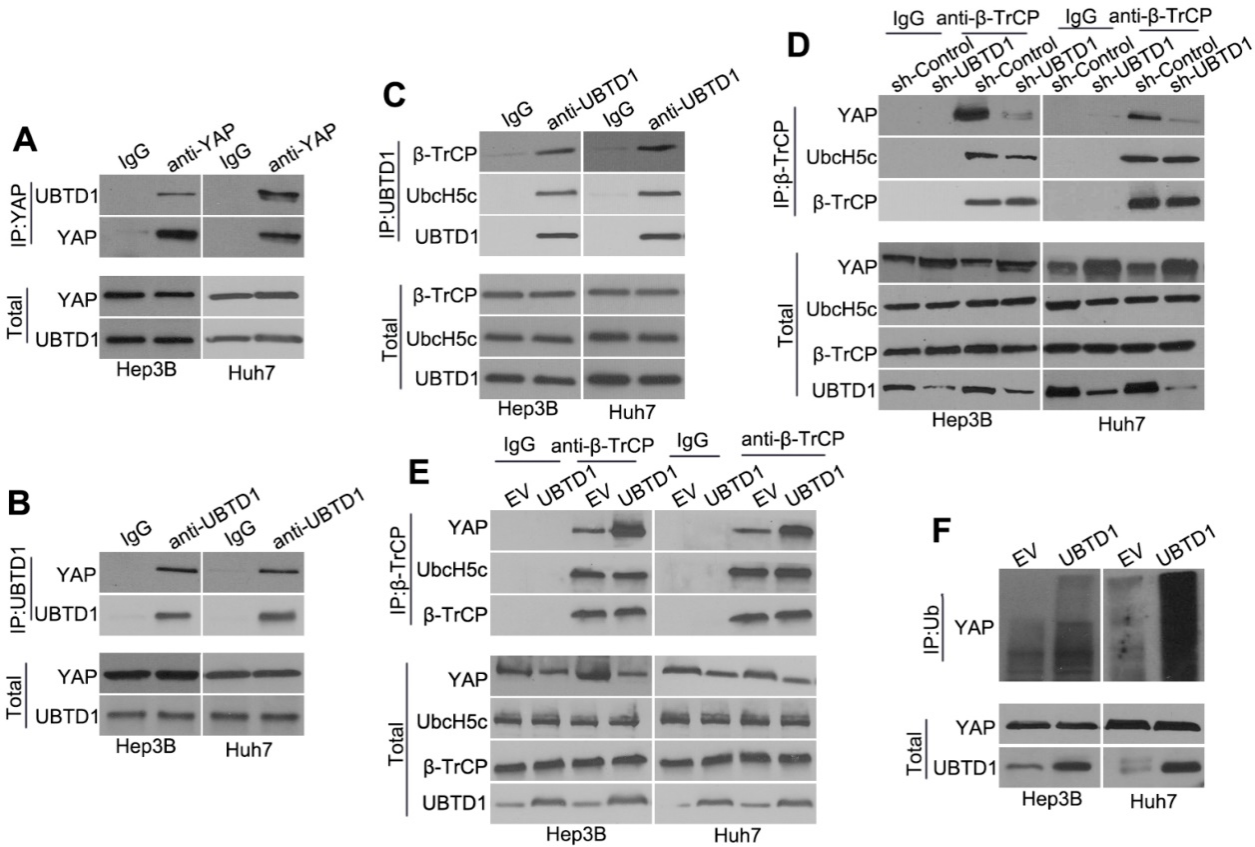
UBTD1 promotes association between YAP and β-TrCP to induce YAP ubiquitylation. (A) Co-IP of endogenous YAP and UBTD1 in Hep3B and Huh7 cells. YAP was used as a bait. The IgG isotype was used as a negative control. (B) Co-IP of endogenous YAP and UBTD1 in Hep3B and Huh7 cells. UBTD1 was used as a bait. The IgG isotype was used as a negative control. (C) Co-IP of endogenous β**-**TrCP, UbcH5c, and UBTD1 in Hep3B and Huh7 cells. UBTD1 was used as a bait. IgG isotype was used as a negative control. (D) Co-IP of endogenous β-TrCP, UbcH5c, and YAP in Hep3B and Huh7 cells. Hep3B and Huh7 cells were transfected with the UBTD1 shRNA. β-TrCP was used as a bait. IgG isotype was used as a negative control. Immunoblot of UBTD1 shows the level of shRNA-mediated depletion. (E) Co-IP in Hep3B and Huh7 cells between endogenous β-TrCP, UbcH5c, and YAP. Hep3B and Huh7 cells were transfected with the UBTD1overexpression vector. β-TrCP was used as a bait. (F) YAP ubiquitination was detected by Western blotting. UBTD1 overexpression markedly promoted YAP ubiquitination.

To investigate how UBTD1 regulates YAP degradation, we performed Co-IP experiments and demonstrated that UBTD1 was associated with YAP (Figure 5A-B). We used Co-IP assays and confirmed the association between UBTD1 and the YAP degradation complex consisting of UbcH5c and β-TrCP, the E3 ligase that is responsible for targeting YAP degradation (Figure 5C). The effect of UBTD1 on the YAP degradation complex was verified by immunoprecipitating β-TrCP, demonstrating that the UBTD1 knockdown drastically reduced the association between YAP and β-TrCP, while UBTD1 overexpression increased this association (Figure 5D-E). These results were functionally validated by performing the YAP ubiquitylation assay. UBTD1 overexpression markedly increased the ubiquitination of YAP (Figure 5F), suggesting that UBTD1 was required for its ubiquitylation. In conclusion, our results indicated that UBTD1 promotes association between YAP and β-TrCP to induce YAP ubiquitylation.

### UBTD1 mediates matrix stiffness-induced effects in HCC cells

**Figure 6:**
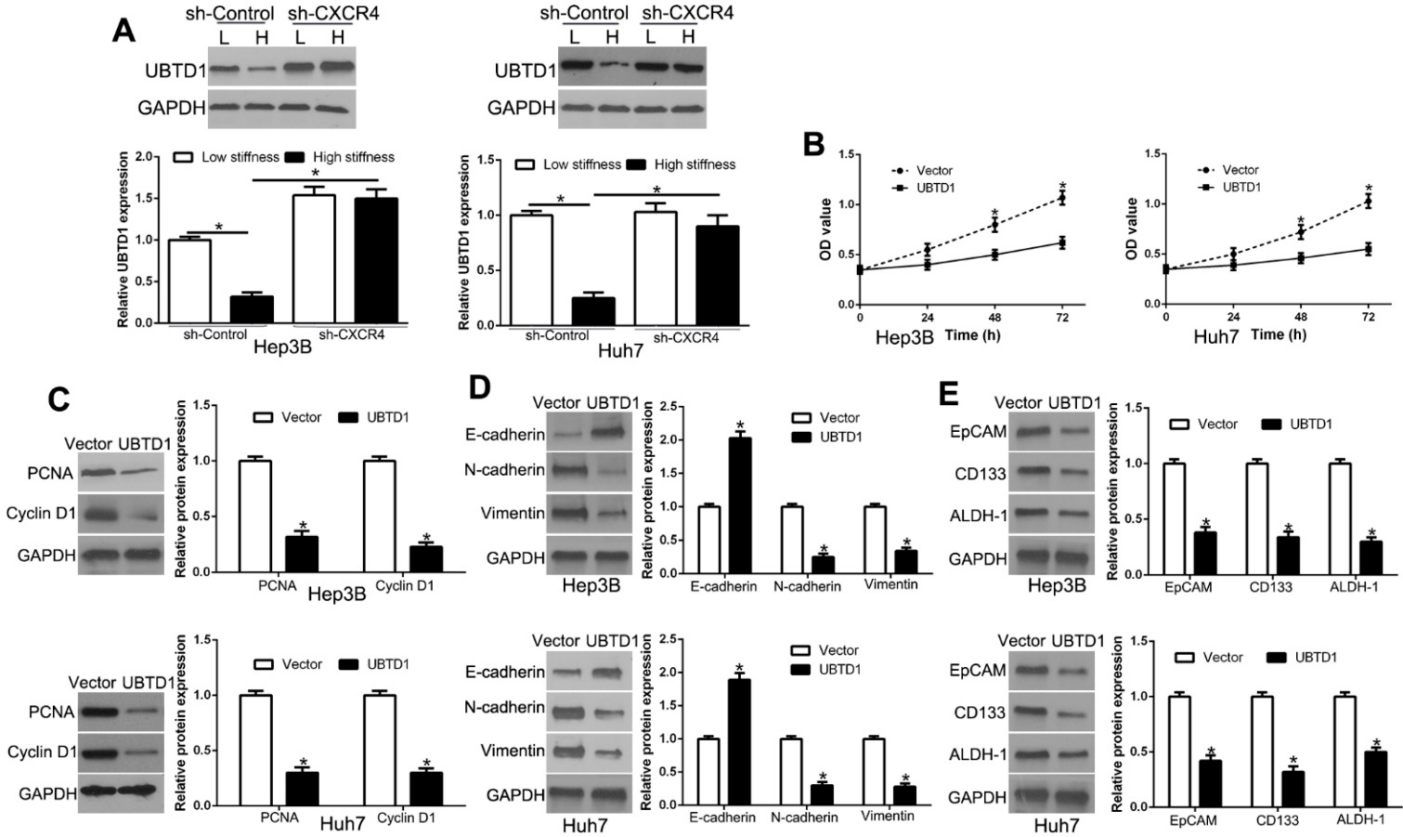
UBTD1 mediates matrix stiffness-induced effects on HCC cells. (A) CXCR4 knockdown reversed high stiffness-induced UBTD1 down-regulation. (B) MTT assay was used to confirm that UBTD1 overexpression inhibited the proliferation of Hep3B and Huh7 cells. UBTD1 overexpression inhibited PCNA and CyclinD1 expression (C), EMT (D), and stemness (E) associated markers in Hep3B and Huh7 cells. *P < 0.05.

To determine whether matrix stiffness-induced UBTD1 down-regulation was dependent on CXCR4, we knocked down CXCR4, which abolished the effects of UBTD1 down-regulation by matrix stiffness (Figure 6A, P < 0.05). When we overexpressed UBTD1, it reversed the effects of high matrix stiffness on proliferation, EMT, and stemness (Figure 6B-E, P < 0.05). Collectively, these data demonstrated that UBTD1 plays an important role in the matrix stiffness-induced effects on HCC cells.

### UBTD1 is down-regulated in HCC and correlates with patient survival

**Figure 7:**
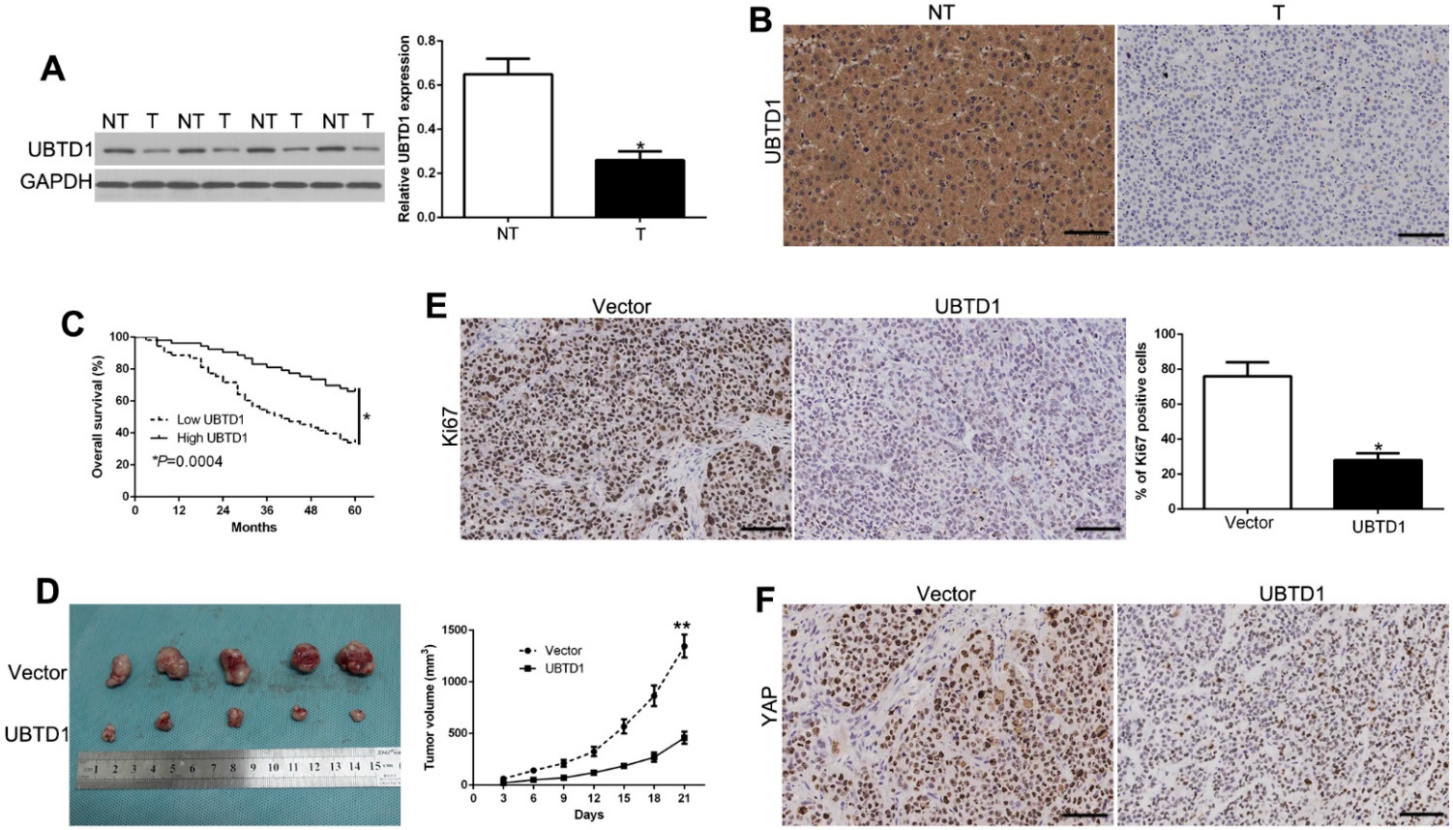
UBTD1 is down-regulated in HCC and correlates with patient survival. (A) Western blot analysis of UBTD1 levels in paired human HCC and matched adjacent non-tumorous tissues. (B) Representative images of IHC staining of UBTD1 in HCC and non-tumor tissues. (C) Overall survival rates of HCC patients in low-UBTD1 and high-UBTD1 groups. (D) Tumor growth curve revealed that UBTD1 overexpression significantly inhibited tumor growth *in vivo*. Tumor nodules were subjected to immunohistochemical staining for Ki-67 (E) and YAP (F). *P < 0.05.

We measured UBTD1 levels in tumors and paired adjacent nontumor liver tissues to validate its role in HCC progression. UBTD1 protein was remarkably down-regulated in HCC tissues compared to the corresponding normal liver tissues (Figure 7A, P < 0.05). Immunohistochemical staining was performed to confirm low levels of UBTD1 protein in primary HCC compared to adjacent normal tissues (Figure 7B). HCC patients were divided into two groups according to the UBTD1 expression to determine its clinical significance in the outcome of HCC patients. As shown in Table 1 (Pearson chi-square test), low UBTD1 was significantly associated with tumor-node-metastasis (TNM), stage (III+IV, P = 0.010), venous invasion (P = 0.010), and tumor size (P = 0.006). More significantly, Kaplan-Meier analysis showed that patients with low UBTD1 had poorer overall survival rates (Figure 7C, P < 0.05).

We also established a subcutaneous tumor model to measure the effect of UBTD1 on HCC *in vivo*. Tumor growth curves revealed that UBTD1 overexpression significantly inhibited the tumor growth of HCC cells in mice (Figure 7D, P < 0.05). Furthermore, UBTD1 overexpression markedly decreased lung metastasis *in vivo* (Figure S7). Next, we evaluated the proliferative rate in the xenografted tissues by Ki67 measurement. As expected, UBTD1 overexpression decreased the number of Ki67-positive cells (Figure 7E, P < 0.05) as well as YAP staining in tissues (Figure 7F). These findings suggested that UBTD1 has significant pathological implications in HCC development.

### *Luteolin* inhibits matrix stiffness-induced CXCR4 signaling pathway and HCC growth

**Figure 8:**
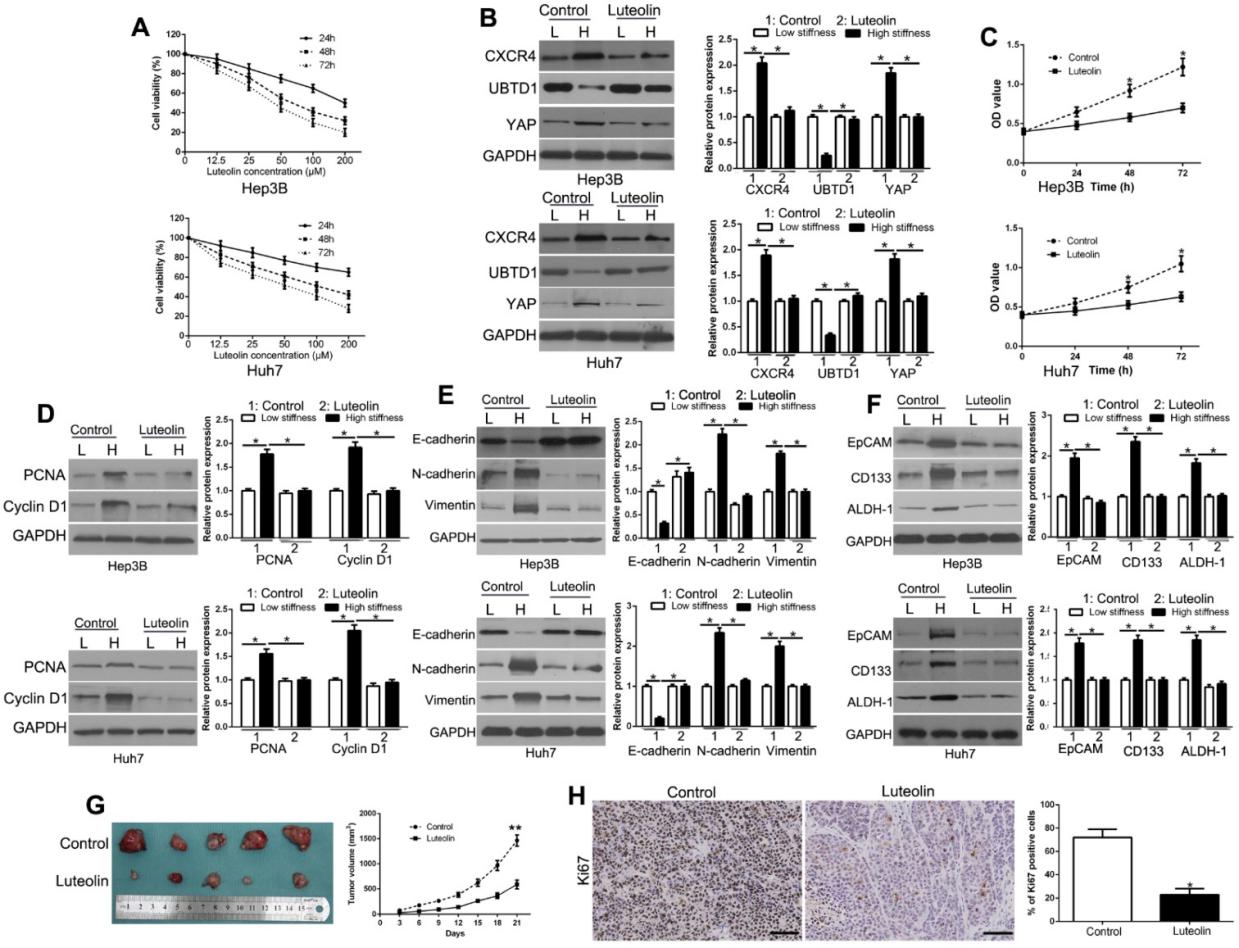
Luteolin inhibits matrix stiffness-induced CXCR4 signaling pathway and HCC growth. (A) Cell survival analysis revealed that luteolin inhibited HCC cell viability in a time- and dose-dependent manner. (B) Luteolin reversed high stiffness-induced CXCR4, UBTD1, and YAP expression. (C) MTT revealed that stiffness-mediated HCC proliferation was abrogated by luteolin. HCC cells seeded on low or high stiffness were treated with luteolin. Stiffness-mediated PCNA and CyclinD1 expression in HCC cells (D), EMT (E) and stemness (F) characteristics were abrogated by luteolin. (G) Tumor growth curve revealed that luteolin significantly inhibited tumor growth *in vivo*. (H) Tumor nodules were subjected to immunohistochemical staining for Ki-67. *P < 0.05.

The above data elucidated the molecular mechanisms underlying the matrix stiffness-induced biological behavior in HCC cells. To investigate new agents for the treatment of HCC, we selected luteolin, which is an abundant flavonoid present in many types of vegetables, fruits, and medicinal herbs, and has been used in Chinese traditional medicine to treat various diseases, such as hypertension and cancers 37. We treated HCC cells with different concentrations of luteolin to examine its inhibitory effects. As detected by the MTT assay, luteolin treatment significantly decreased cell viability in a time- and dose-dependent manner (Figure 8A), which was consistent with previous studies. Based upon the MTT assay results, we chose 50 μM concentration of luteolin for subsequent experiments and found that it reversed the effects of matrix stiffness-induced CXCR4 signaling activation (Figure 8B, P < 0.05). Our data further showed that luteolin reversed the effects of high matrix stiffness on the proliferation, EMT, and stemness of HCC cells (Figure 8C-F, P < 0.05) and inhibited HCC growth *in vivo* (Figure 8G, P < 0.05). Also, Ki67 staining further confirmed that luteolin blocked the proliferation of HCC cells *in vivo* (Figure 8H, P < 0.05). Luteolin also regulated CXCR4/UBTD1/YAP expression in the subcutaneous tissues (Figure S8). Collectively, these data demonstrated that luteolin has anti-cancer effects on HCC cells.

## Discussion

Liver cancer is a neoplasm with high incidence and mortality that carries a dismal prognosis. Previous reports showed that pathophysiological increases in matrix stiffness, which are encountered in fibrotic and cirrhotic livers, promoted HCC development and progression 38. Although the significant correlation between matrix stiffness and HCC has attracted much attention from oncologists in the last decades, the detailed mechanism by which matrix stiffness mediates its effects on HCC progression remains largely unknown.

In the present study, we demonstrated that matrix stiffness profoundly alters the phenotype and behavior of HCC cells, including proliferation, EMT, and stemness characteristics. Chemokine receptors are the major regulators of cell trafficking and adhesion. In particular, CXCR4 has been implicated in both tumor cell dissemination from the primary site and intravasation via trans-endothelial migration. The interaction of CXCR4 and its ligand SDF-1 promotes HCC metastasis, proliferation, and angiogenesis 39. Here, we identified, for the first time, that CXCR4, as a critical intracellular signal transducer, relays matrix stiffness signals to control mechano-sensitive cellular activities through YAP signaling. It has been reported that CXCR4 density, organization, cholesterol content, and matrix stiffness affect the unbinding force of CXCR4 in breast cancer 27. CXCR4 was significantly upregulated with increased matrix stiffness and mediated the biological effects of stiffness on HCC. Previous studies showed ECM rigidity, cell tension, and changes in cell geometry mediate cell contractility, which, in turn, activates a YAP-dependent mechano-response independent of the Hippo cascade 40.

Herein, we confirmed that stiffness induced YAP nuclear targeting and YAP-dependent transcription of target genes. YAP/TAZ is mechano-sensitive transcription coactivators. They reside in the cytoplasm on a soft substrate and translocate into the nucleus by stiffness. In cancer biology, the crucial role of YAP pathway has been widely emphasized at many stages of cancer progression, including proliferation, invasion, and metastasis and, therefore, deregulation of YAP signaling is oncogenic in humans 41. In this study, we identified UBTD1, a previously uncharacterized ubiquitin-like protein, as an important player controlling the mechanical properties of cells. UBTD1 was markedly decreased with increasing matrix stiffness. This phenotypic alteration reflected some major modifications in the matrix stiffness. YAP pathway has been reported to be versatile upon mechanical stimulation. YAP could be activated by cytoskeleton remodeling and ROCK signaling in response to matrix stiffness 27. We demonstrated that UBTD1 knockdown induced robust activation of YAP signaling. Concomitantly, we noticed that YAP was strikingly higher upon the UBTD1 knockdown induced by matrix stiffness, suggesting a possible role of UBTD1 in YAP protein stability.

It has been reported that the β-TrCP E3 ubiquitin ligase catalyzed YAP ubiquitination, ultimately leading to YAP degradation. Our data provided converging evidence indicating that UBTD1 was associated with YAP degradation complex and participated in its ubiquitylation by β-TrCP. We also demonstrated that UBTD1 was decreased in human HCC tissues. UBTD1 expression was highly correlated with poor pathological features, including the TNM stage, venous invasion, tumor size, and overall survival. Moreover, UBTD1 overexpression inhibited proliferation, EMT, and stemness. We further confirmed that the natural medicine, luteolin, inhibited the matrix stiffness-induced CXCR4-mediated YAP signaling pathway, proliferation, EMT, and stemness (Figure S9) thus identifying luteolin as a promising therapeutic agent for HCC.

In summary, we have characterized CXCR4 as a new mechano-response linked to the matrix stiffness. The intricate UBTD1 down-regulation controlled YAP signaling and participated in YAP degradation by modulating its ubiquitylation. Significantly, luteolin suppressed matrix stiffness-induced CXCR4/YAP signaling. These results suggest that luteolin is a potential anti-cancer agent for the treatment of HCC.

## Supplementary Material

Supplementary figures.

## Figures and Tables

**Table 1:** Clinical correlation of UBTD1 expression in HCC (n = 106).

Clinical parameters	Cases (n)	Expression level		*P* value (* *p*<0.05)
		UBTD1^high^(n=53)	UBTD1^low^(n=53)	
Age(years)				
<60 years	64	31	33	0.691
≥60 years	42	22	20	
Gender				
Male	81	39	42	0.492
Female	25	14	11	
Tumor size (cm)				0.006*
<5cm	58	36	22	
≥5cm	48	17	31	
Tumor number				0.164
solitary	91	48	43	
multiple	15	5	10	
Edmondson				0.164
Ⅰ+Ⅱ	24	15	9	
Ⅲ+Ⅳ	82	38	44	
TNM stage				0.010*
Ⅰ+Ⅱ	83	47	36	
Ⅲ+Ⅳ	23	6	17	
Venous infiltration				0.010*
Present	9	1	8	
Absent	97	52	45	
AFP				0.353
<400ng/ml	24	14	10	
≥400ng/ml	82	39	43	
HBsAg				0.727
positive	97	49	48	
negative	9	4	5	

HCC, hepatocellular carcinoma; AFP, alpha-fetoprotein; TNM, tumor-node-metastasis. *Statistically significant.

## Abbreviations

CXCR4: C-X-C chemokine receptor 4
UBTD1: ubiquitin domain-containing protein 1
YAP: Yes-associated protein
HCC: hepatocellular carcinoma
EMT: epithelial-mesenchymal transition
IHC: immunohistochemistry
shRNA: short hairpin RNA
IF: immunofluorescence
WB: Western blot
ECM: extracellular matrix
DMEM: Dulbecco's modified Eagle's medium
