# Supplementary material for: CXCR4 mediates matrix stiffness-induced downregulation of UBTD1 driving hepatocellular carcinoma progression via YAP signaling pathway
Source: Theranostics. 2020 Apr 27;10(13):5790–801. doi: 10.7150/thno.44789 (PMC7255012; doi:10.7150/thno.44789)
Supplement: Supplementary file 1 — Supplementary figures. [file thnov10p5790s1.pdf]

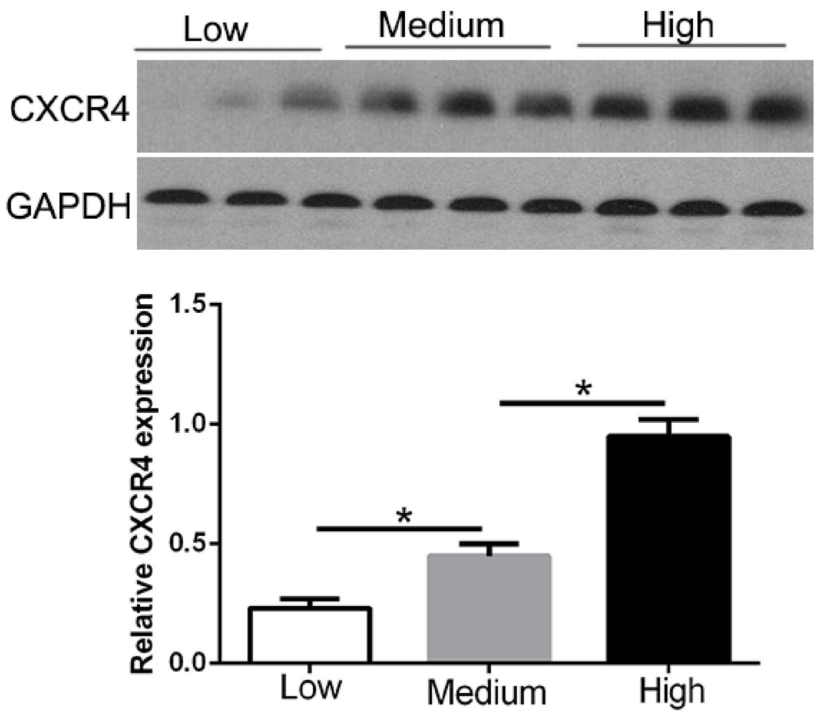

Figure S1. Western blotting showing high expression of CXCR4 in HCC tissues with high and medium stiffness background as compared with that of normal stiffness background. *$P < 0.05$.

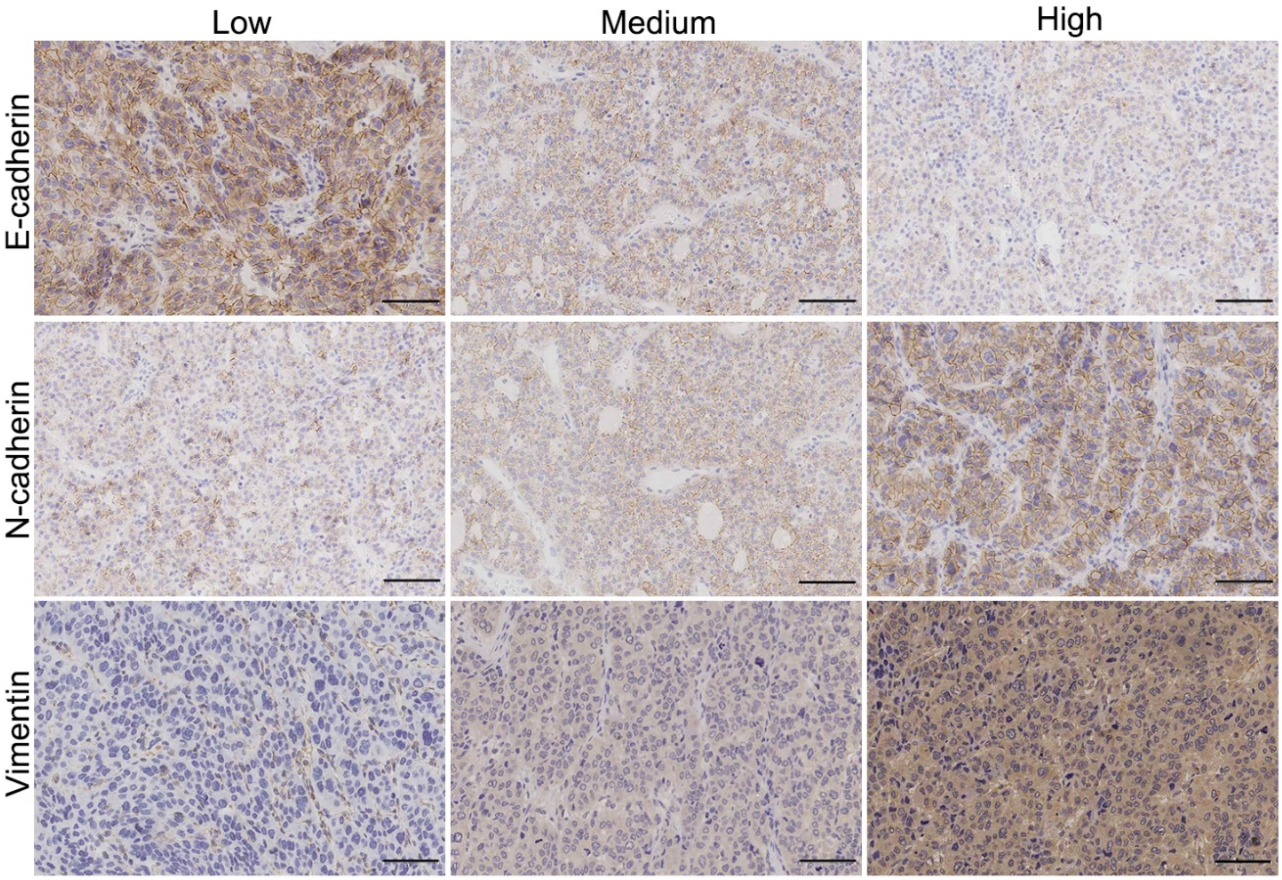

Figure S2. Expression levels of EMT-related proteins in HCC tissues with normal, medium, and high liver stiffness backgrounds.

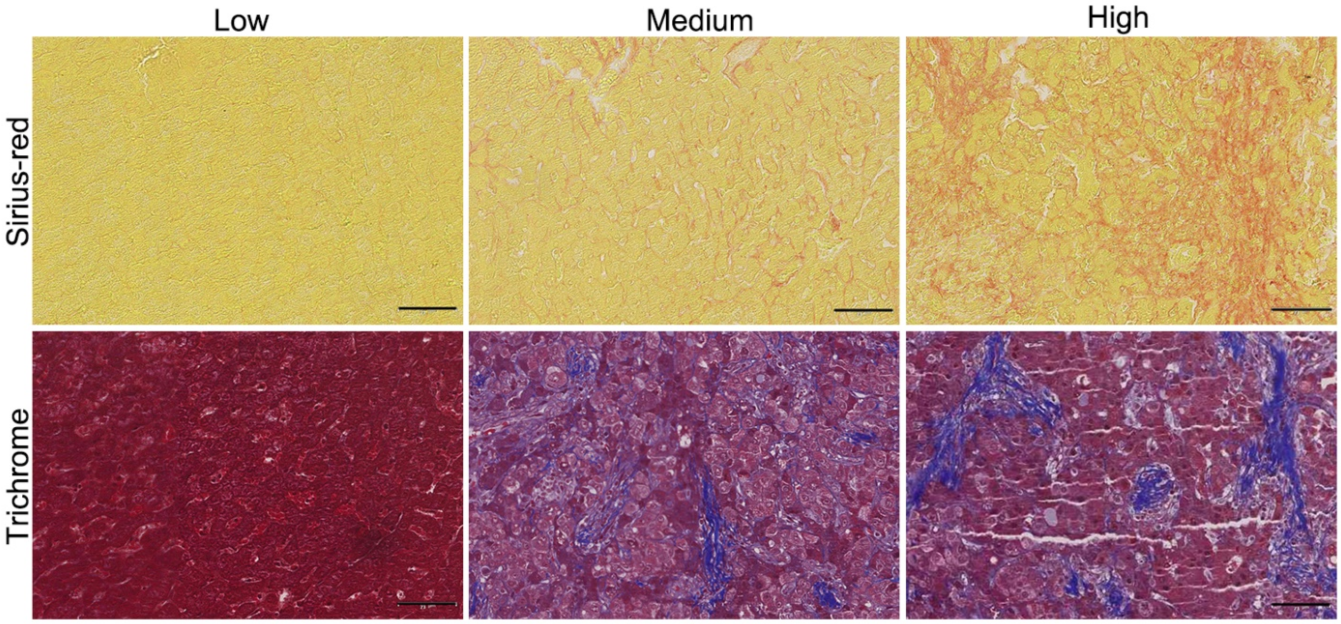

Figure S3. Sirius red and trichrome staining to show the extracellular matrix in normal, medium, and high liver stiffness backgrounds.

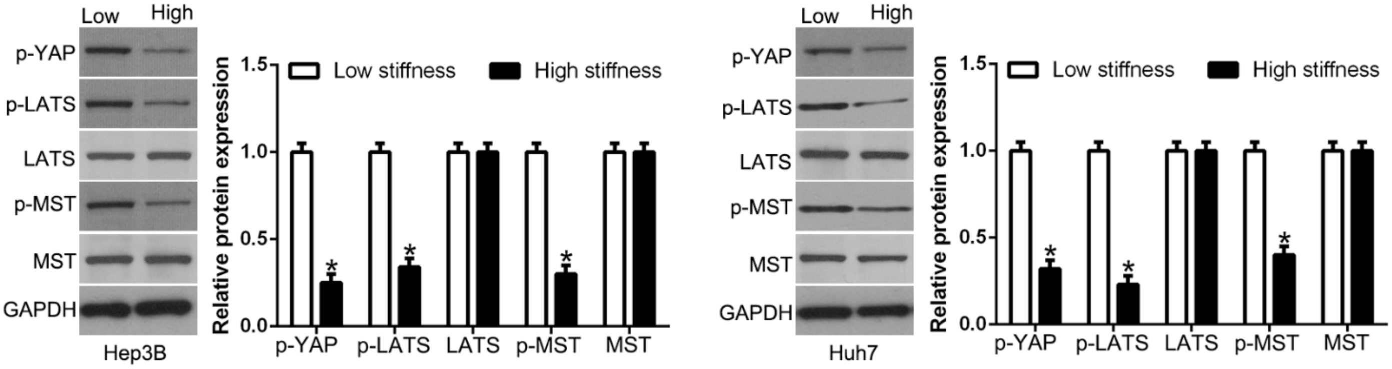

Figure S4. Western blotting showing that matrix stiffness regulated the phospho-YAP, MST, phospho-MST, LATS1, and phospho-LATS1 status in HCC cells. *P < 0.05.

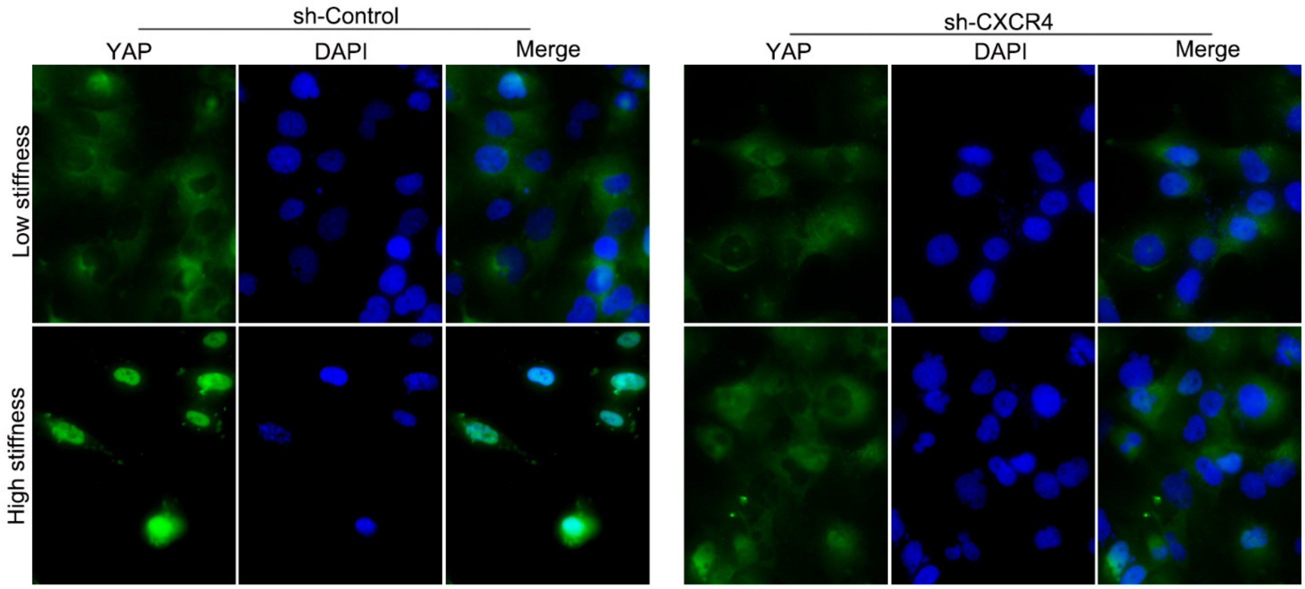

Figure S5. Stiffness-mediated YAP upregulation and nuclear accumulation were abrogated by CXCR4 shRNA in Huh7 cells.

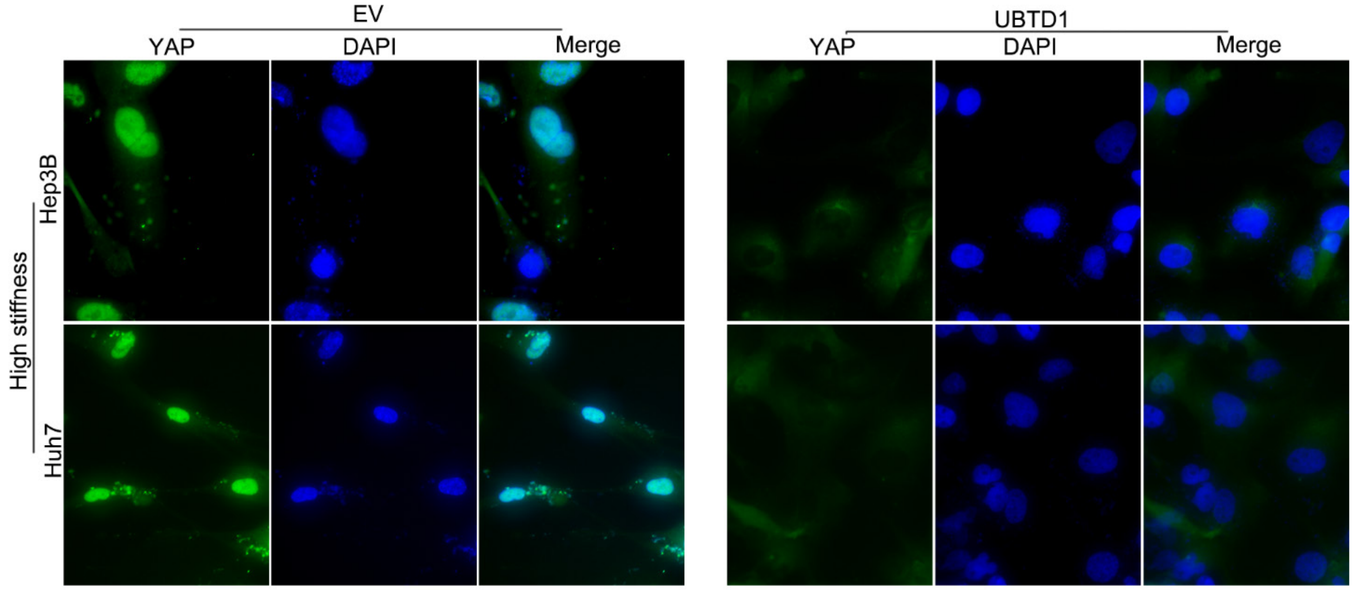

Figure S6. UBTD1 overexpression changed the localization of YAP in high stiffness.

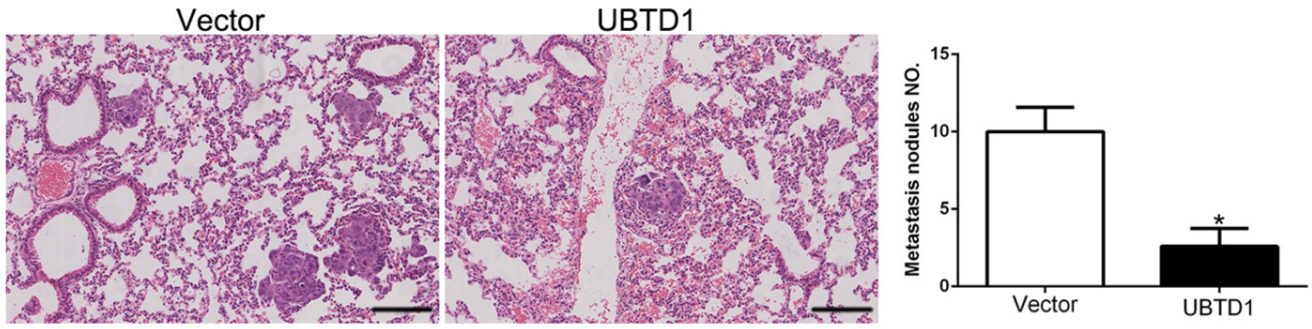

Figure S7. UBTD1 overexpression markedly decreased lung metastasis in vivo. *P < 0.05.

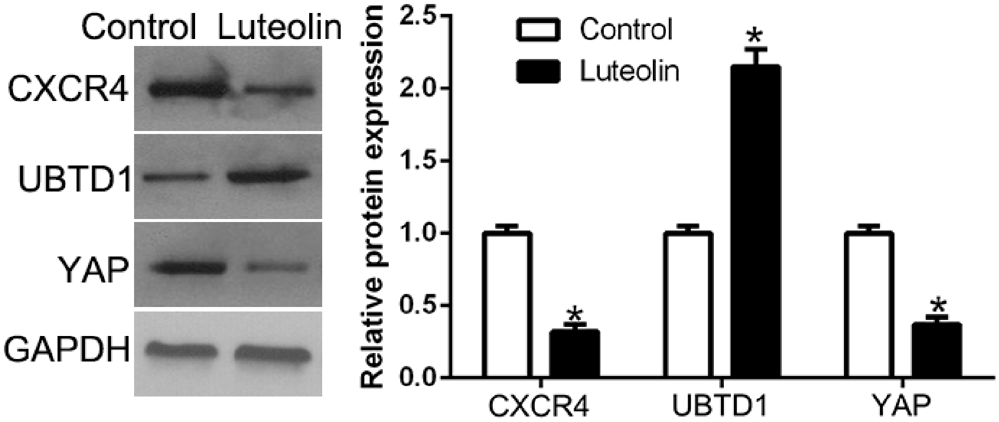

Figure S8. Luteolin regulated CXCR4/UBTD1/YAP expression in the subcutaneous tissues. *P < 0.05.

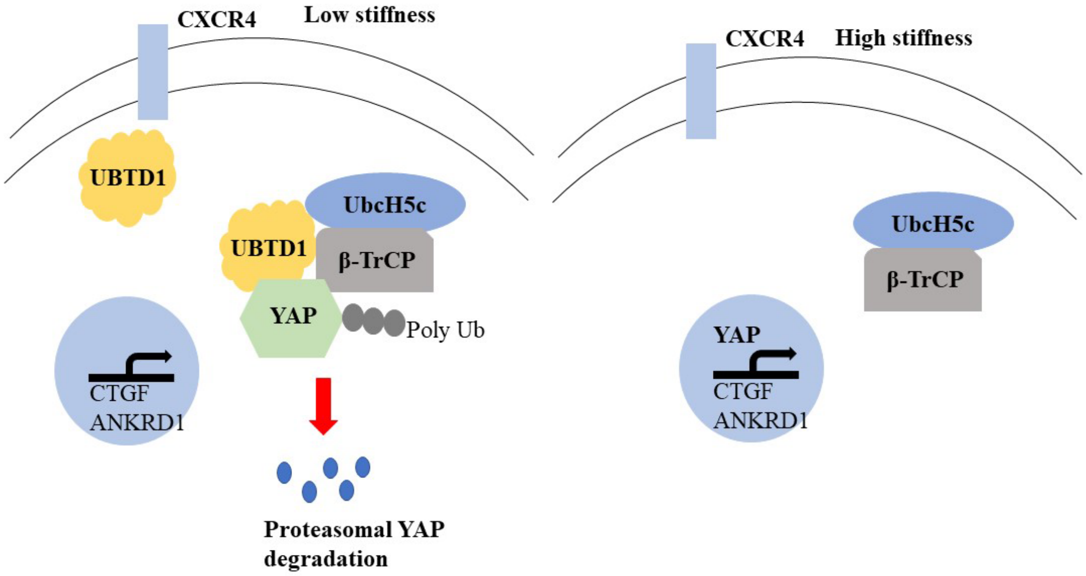

Figure S9. Schematic showing that CXCR4 is a mechano-transducer that relays extracellular mechanical signals to induce UBTD1 down-regulation, which decreases YAP ubiquitylation degradation, resulting in activation of YAP targeting genes. UBTD1 promotes the interaction of YAP with its E3 ubiquitin ligase β-TrCP, and decreases YAP ubiquitylation to trigger YAP activation and its downstream signaling. Matrix stiffness acts through CXCR4 and its downstream UBTD1 to modulate a YAP/TAZ-mediated mechano-responsive signaling pathway.
